# Hospital and Institutional News

**Published:** 1919-04-19

**Authors:** 


### HOSPITAL AND INSTITUTIONAL NEWS.

### EXPERIMENTS ON DOGS.

At a joint meeting of the sections of the B.M.A., Sir William Osler moved a resolution to the effect that the profession heard with dismay of the probability of a Bill passing the House of Commons to prohibit experiments upon dogs. Such action was calculated to hinder the progress of medicine and to render Britain alone unable to continue progress in certain departments of medical research. Colonel Martin, in seconding, stated that there were two reasons for this introduction, namely, a want of appreciation of what this type of experiment had meant to medical advance in the past and a misapprehension as to what happened to the dogs in the laboratories. Men were frequently experimented upon—they nobly volunteered; but this could not be expected in every instance. He had never seen anything done to a dog in the laboratory that he would object to being performed upon himself if the only alternative were death in a lethal chamber. We feel that there are, of course, two sides to the question and that scientifically the research workers can put forward a powerful, probably to them overwhelmingly strong, case. Certainly, if the doctors' claims of obstruction be even approximately true, the matters are of such importance that the Bill should not, under any circumstances, be rushed through. The situation should be thoroughly discussed in committee with the aid of opinions from experienced and practical men. We believe that the issue does not, at the moment, officially rest with people in every case qualified for the task, and hope to see a thoroughly fair and representative discussion. If the scientific claims are just, then, with Sir William Osler, we say that we give best to none in our love for the dog, but our love for our fellow men must rank superior to our affections for a dumb animal however greatly we appreciate his comradeship.

### THE INFLUENZA DISCUSSION.

No great finality appears to have been as yet reached on the question of the cause of the recent "influenza" epidemics. Opinions recently expressed at South Kensington still leave us to decide between the minute filter-passing germ and the previously known catarrh-producing organisms. Some of the facts and figures quoted give, however, a striking illustration of the intensity of a series of epidemics, the like of which has never been known in the history of medicine. The Press has given much space to description of the ravages among the civil population, but when we hear that in six weeks the B.E.F. in France had just on 50,000 cases with well over 3,000 deaths it brings home the truth of statements that the disease has been "more virulent than the plague." Every army in the field, allied and enemy, has suffered to an extent not yet fully realised. At sea it has been even worse. There were at least 80,000 cases of influenza in the Navy; in one ship's company of 718 men no fewer than 662 were attacked, while the acuteness of the transport trouble with both our own and American troops is illustrated by the instance of one vessel, carrying 2,800 men, which put about on the third day and landed 660 cases. The voyage was then continued, but 1,000 more were attacked. Many died at sea or were landed in a dying condition. On the whole, however, opinion appears to be in favour of finding an explanation in the abnormal conditions of humanity in a war-stricken world having given opportunity for remarkable activity on the part of previously known catarrh and pneumonia-producing germs, and that we have no need to look for a new bacteriological type.

### A POST-WAR PROBLEM.

Official announcements of figures at the national health conference of the Faculty of Insurance held last week at the Central Hall, Westminster, are in many instances of great interest to those concerned in the various organisations which aim to relieve some of the health difficulties imposed by the war. To disabled soldiers and sailors, already over 12,000 pensions for officers and nearly 600,000 for men have been granted, and 20,000 are now being made each week to those being demobilised. There are many more to come through this channel and also many for the men still under treatment in home military hospitals. At armistice time, there were over half a million men in hospital at home and abroad; to-day, the number is reduced to about 200,000. Estimates state that there will be at least 700,000 temporary pensioners, of whom a considerable, but at present unknown, number will become entitled to permanent aid. The Ministry of Pensions is taking over orthopœdic hospitals from the Army and establishing clinics in the surrounding country. Nervous disability, to which we referred a short time ago, is to receive the same centralised consideration, and with regard to men requiring trade education or training, there are now found in these classes 24,000 men who have lost a limb by amputation, 128,000 who have suffered complete disablement in a limb, 60,000 suffering from chest complaints, 54,000 with heart trouble, and nearly 12,000 were either deaf or blind. The Ministry of Pensions has trained, or is training, 24,000 men, but new schemes are required and great extensions needed. At present it will confine its actions to training men during the time they still require medical care and attention. The Ministry of Labour has the subsequent phases under its control, and they form a problem of very great magnitude. Assistance in any form will be given in the best of good causes.

### NEW MEDICAL SUPERINTENDENT.

Dr. R. C. Harkness, M.B. (Edin.), M.R.C.S. (Eng.), L.R.C.P. (Lond.), F.R.C.S. (Eng.), has been appointed medical superintendent of the Bermondsey Infirmary, vacant through the death of Dr. E. S. Bell. The applications were first of all considered by a committee consisting of Sir Cooper Perry, of Guy's Hospital, Dr. R. King Brown, Medical Officer of Health for Bermondsey, and Dr. A. Salter, a member of the board. This committee submitted eleven candidates for the consideration of the guardians, who unanimously appointed Dr. Harkness. He is at present medical officer at Harton Hospital, South Shields, which position he has held since May, 1915. Positions previously held included first house surgeon of the Gynæcological Wards at Edinburgh Royal Infirmary, house physician at Manchester Royal Infirmary, house surgeon at Wolverhampton General Hospital, senior resident medical officer at the London Temperance Hospital, and senior house surgeon at Leicester Royal Infirmary.

### THE PRINCE AND THE PROLETARIAT.

The Prince of Wales is following the example of his great predecessors in making himself the expression of the national conscience by a series of visits to slums, for these visits symbolise our open admission of these social sores to which we have far too long shut our eyes. The slum now receives the visit which the splendid mansion alone used to expect, because the worst is now seen to be of equal importance. These visits are the development of those paid by Royalty to hospitals, because hospitals were, and are, the first social attempt at better things. But we must not confine our attention to our best endeavours. We must face no less resolutely all that remains to be done. We know what the Royal Family have been able to do for hospitals. May we not hope that their work will be equally fruitful in a less attractive field?

### THE LEISURED WORLD OF LABOUR.

It is not often that members of committees are charged with negligence in routine work, except in industrial districts, for the leisured world of Labour seems to be the only section of society which has time to waste on committee meetings, or an appetite for the discussions which they involve. That this, if anything, is an understatement of Labour's weakness is apparent from the fact that Labour, and Labour alone, favours the inflation of committees, and consequently trebles the time of the meetings and does more to prevent quick decisions than anything else. The absurdity becomes ludicrous when the members of a committee of 40 or 70 are charged, as sometimes happens, with irregular attendance. For the best service the member of a huge committee can make is to absent himself from its meetings, partly to reduce its numbers, and partly to emphasise the fact that a committee is useful in inverse proportion to its size. We commend these truisms to the criticasters who lately alleged the enormity of "negligence" against certain members of the committee of Poplar Hospital.

### PORTS AND DISEASE.

There has lately been much emphasis laid upon the increased importance and possibility of disease entry to this country and the part played by the port sanitary authorities in its prevention. The Government, it was stated at a recent meeting of the Dover Corporation, are showing an active appreciation of the facts by providing the expenses of the port authorities incurred in carrying out such work. Apart from the fact that local prevention is a consideration made directly for the whole nation, and should therefore be a national and not a local charge, we are glad to see this action as tending to improve and extend medical and sanitary work which is of vital importance to the community.

### THE FUTURE OF QUEEN'S HOSPITAL, BIRMINGHAM.

The big scheme for the reorganisation of Birmingham hospitals outlined by Mr. Barling, and lately summarised in The Hospital, is part of a general tendency in Birmingham, where all the hospitals require extension and where each has its own difficulties of money or of site. For instance, having dealt with the Birmingham General Hospital, let us turn to the Queen's. Large extensions have been considered for some time, but the site offers as much difficulty as does the shortage of funds. Mr. Boultbee Brooks, the chairman of the Queen's Hospital, lately pointed out that 1,000 more beds were needed in Birmingham, or, in other words, that the existing number should be doubled. Mr. Brooks hinted, in fact, that the building of such a hospital would have to be considered by his own hospital's committee, because the present building could not be sufficiently enlarged, and argued that with modern means of communication the site of the proposed hospital need not be in the centre of the city. He added that the number of soldiers among the patients should encourage the public to regard the scheme, if approved by the committee, as a War Memorial. On the other hand, though new hospitals will not normally be so expensive as the old, yet Mr. Brooks favours State subvention, somewhat on the lines adopted in Australia, which preserves the voluntary system by making State grants proportional to the amount voluntarily subscribed. Mr. Brooks' large views deserve every support that a go-ahead city can give them. Mr. Neville Chamberlain, M.P., one of Birmingham's foremost citizens, has also been making some observations on what he describes as "the danger of the bankruptcy of the voluntary system." Voluntary institutions, he pointed out, were already receiving, through local authorities, assistance from the State in respect of special services, and he thought it was to an extension of that system that we must look for the future of voluntary hospitals.

### EFFORTS ENDED, AND IN PROGRESS.

With the closing of the American Hospital for English Soldiers, at Highgate, an institution definitely ceases with the war. But the same is not to be the case with the South African Hospital at Richmond, which, after its present work shall have become finished in the future, is to become an orthopædic hospital under, we believe, Government patronage. Of the 1,000 odd cases at present at Richmond, about one quarter are not South Africans, and the bungalow plan on the present site has proved so successful that the idea of removal has been given up. The present staff will be reduced slowly. The training-centre will probably be permanent and the whole institution should be an important addition to the hospitals in the Old Country.

### A PAY HOSPITAL FOR FREEMASONS.

Among developments towards hospitals for paying patients must be mentioned that instituted by the Freemasons. The craft has been conducting three hospitals for the wounded during a considerable part of the war. These are the premises in the Fulham Road, formerly the Chelsea Hospital for Women, Fulham Palace, and another institution near Reading. The premises in the Fulham Road were acquired with the ulterior object of becoming a hospital for patients of limited means connected with Freemasonry. Such an institution was contemplated before the war, and a committee, of which the present Lord Mayor is the Treasurer, was supported by an influential medical advisory committee. The institution will have some forty beds, and the charges proposed would not be more than sufficient to cover the cost of nursing and food. It should thus fill an intermediate place in the hospital world to the benefit of Freemasons and their families. The institution is already equipped. It has an *x*-ray installation, and an electro-therapeutical department of which any small hospital might be proud.

### THE PERMANENCE OF DISPENSARIES.

One of the delusions which ushered in the Insurance Act was that there would be no place, after its passage into law, for the dispensaries. It is striking, therefore, to read how the attendances at the Liverpool Dispensaries continue to increase, and how these institutions are stated to fill the gap which waits for those who stand between the Scylla and Charybdis of the panel and the Poor Law. It is also remarkable how much they do and have been doing for mothers and infants before this work became fashionable under the name of "child welfare." The dispensaries will continue when the Insurance Act has been so amended as to have lost all the "refreshing fruits" which have proved so rotten in experience.

### WELLINGTON COTTAGE HOSPITAL.

The cottage hospital at Wellington, near Wolverhampton, has changed its name so as to include the word "district." The change means that it will cease, in the public mind, to be regarded merely as a monument to the late Mr. J. C. Crump, and become, by an alteration in the rules, more accessible to poor patients. The charge for patients is to be a flat rate of 3s. 6d. a week, and every recommendation is to hold good for a month.

### THE CHEPSTOW HOSPITAL.

What are called Government scandals have the advantage of reminding foolish people that State control is not the synonym of "efficiency"—that misunderstood term which covers a multitude of sins. Chepstow has become a byword of what the State's notion of "efficiency" may mean; and the unrequired hospital built there in connection with the wretched national shipyards, will either have to be scrapped, or, to save the face of the Government, the plan for treating orthopœdic cases just outside Cardiff will be interfered with. Chepstow is inaccessible except by aeroplane, and air-ambulances are not very numerous at present. To say the best they are scarcer even than taxi-cabs in London. According to the *British Medical Journal*, the Chepstow buildings have been offered to the Monmouthshire County Council for that last refuge of the Government in search of a "subject," the mental defective. If some members of the Cabinet were transferred there from Chepstow it might still further commend this remedy for a bad job. But Cardiff. and its great medical school, must not be allowed to suffer to screen the folly of those in power.

### FREE BEDS FOR A PENNY A WEEK.

When the armistice brought the work of the local Prisoners of War Committee to a close, the committee and Mr. W. Whyers decided to assist Boston Hospital, Lincolnshire. They therefore proposed that free beds should be provided for subscribers of one penny a week. Already 500 men on the Great Northern Railway and between 200 and 300 men employed by firms in the town, together with the large majority of those at work at the dock, have enrolled themselves as subscribers. Two railwaymen have been enjoying free beds, for the house committee have approved of the scheme and recommended that every subscriber of one penny a week, whether an employer, an employee, a man, a woman, or a child, shall be entitled, on a doctor's recommendation to the use of a free bed, always providing that a bed is vacant. To the 20 beds at present in use, it is hoped to add the ten beds which were closed some while ago. Mr. Whyers' committee collects the subscriptions quarterly from the firms which take them by consent from the weekly wages. A curious gift has also been received—namely, a donation of £150—from the parish of Fishtoft, on condition that for four weeks every year in perpetuity the parish shall have the use of a free bed in the hospital. It is hoped that other parishes will give similar gifts on similar conditions.

### THREE-FIGURE ANNUAL SUBSCRIPTIONS.

Why should annual subscriptions be limited to a handful of guineas when a hospital would benefit perhaps more than in any other way from the interest (by way of subscription) from the large donation? How can annual subscriptions be encouraged if the request is limited, as it naturally is, to the paltry figure of a guinea or two? These questions seem to have been asked of herself by Miss Florence Cory, of Duffryn, Cardiff, with the result that she has offered King Edward VII.'s Hospital an annual subscription of £100 if thirty-nine other people will give the same. This shows financial imagination, and if her enterprise is successful (as who can doubt in such a Babylon of wealth as Cardiff?), Miss Cory will have the satisfaction of knowing that she will have done more for annual subscriptions as a hospital policy than any who can be named in company with her. For these things are largely matters of fashion, and what every hospital secretary has so far failed to do Miss Cory has almost accomplished—namely, to make annual subscriptions the most attractive part of revenue. All this, too, by a stroke of the pen. So powerful is a pen when there is a mind behind it.

### THE NORTH-EASTERN HOSPITALS' ASSOCIATION.

The lately formed North-Eastern Hospitals' Association is, we hope, another instance of that decentralisation and local activity which alone can keep the tendency to bureaucratic dictation from Westminster within bearable bounds. If the Association does its proposed work of acting as a northern bureau of information to the Ministry of Health, there is some chance that the multifarious activities of the latter may be partly delegated to it, which would be a great advantage to all concerned. Mr. R. Orde, the house governor of Newcastle Infirmary, should be of immense aid to the Association, and if he and Dr. Kerr, the Medical Officer of Health for Newcastle, cannot check the encroachments of Whitehall, no one can. Though formed at the eleventh hour, the new Association deserves every help from the hospitals of Durham and Northumberland.

### THE SHAPE OF APPEALS.

Rather a good leaflet has been issued by the Great Northern Central Hospital. The secretary, Mr. Gilbert Panter, has had it so ingeniously folded that each of its ten sides, which look at first like four, gives a different piece of information. Twelve answers are provided to the question " why " the reader should help. There is just enough temptation to unfold each rim to beguile a stranger's attention and to delay the leaflet from the wastepaper basket until the gist of it shall have been read. This suggests that more attention should be paid to the actual shape of appeal-papers than is given at present. For the better appeal-writing is practised the more numerous will appeals be, and the more numerous they are the less will each invite attention. The matter and its presentation in words have been fairly well studied. But what is wanted now is first a more frequent use of the art of the cartoonist and the illustration; secondly, a closer study of the original shapes for leaflets; and thirdly, a record of the more ingenious appeals of the past. Happy ideas are too often used and forgotten, or buried in a careful secretary's scrap-book. Will the wise man who has kept one pass on a selection of its contents to us?

### TEMPORARY COTTAGE HOSPITALS.

The Whitstable Cottage Hospital Committee has arranged with Mrs. Spender, of Tankerton Hospital, to establish a temporary cottage hospital. Originally, strange to say, it was proposed to limit the institution to men and boys, but, thanks to Dr. Witney, it has now been decided to provide a ward for seven women, and to accommodate the men elsewhere in the building. It has wisely been decided to keep the expenses of the temporary hospital as low as possible, so that, as Mr. Hopkins has urged, the fund for the future permanent hospital may be as large as possible. Thus the temporary hospital will not cost more than £600 for the first year, and it is hoped to set the fund on its feet by an appeal by letter to every householder in the town and district.

### THE LION HOSPITAL AID SOCIETY.

One of the less well-known hospital funds is that of the Lion Hospital Aid Society, which disburses annually some £1,500, and last year presented an ambulance to the London Hospital. Another instance of the power of heredity in the Voluntary System is seen in the case of the fund's President, Mr. A. G. Cohen, who, like his father and his grandfather, has for many many years been actively associated with it. Though Mr. Cohen failed to be elected for the constituency at the past election, he had the satisfaction of hearing that his successful rival was celebrating his triumph by the receipt of a mass of correspondence from the electors so enormous as to prevent it being read and dealt with promptly. This suggests that the real satisfaction in life to a busy man comes rather from getting a piece of work quietly through than from being the butt of unknown correspondents, attention to whom involves more time than the result achieved may justify. Hospital work is not much in the limelight, but there is no finer field for a man with a conscience and brains.

### A WORD OF WARNING.

While every effort should be made to convert to the uses of peace those local war collections, such as that for pensioners, we must not forget to revive also those hospital collections which have lapsed during the war. A case in point is that of the Monthly Penny Fund for Hereford General Hospital, which badly needs volunteers to collect from districts where the fund has been suspended during the past few years. It cannot be too often repeated that the hospitals which will have most to gain, and most to lose, from the coming Minister of Health, will be those whose finances are least dependent on his department, and which therefore will remain the least subject to the hampering conditions under which, necessarily, that help will be given.

### CINEMAS IN MENTAL HOSPITALS.

While much has been heard of the educational possibilities of the cinematograph, its more serious uses have hardly been the subject even of experiment. Here, as elsewhere, vulgarity holds the field, for vulgarity is the only thing which is wholly democratic. Perhaps hospitals once more will lead the way to better things. It is, or should be, encouraging to learn that the Asylums Committee of the Metropolitan Asylums Board has suggested the purchase of two more cinemas to "entertain and instruct" the patients and the staff. The phrase is a trifle ominous, for unless instruction is entertaining and entertainment instructive either fails to receive its reward. The separation of the two is the real mistake.

